# Bodily self-recognition and body size overestimation in restrictive anorexia nervosa: implicit and explicit mechanisms

**DOI:** 10.3389/fpsyg.2023.1197319

**Published:** 2023-07-14

**Authors:** Marianna Ambrosecchia, Martina Ardizzi, Elisa Caterina Russo, Francesca Ditaranto, Maurizio Speciale, Piergiuseppe Vinai, Patrizia Todisco, Sandra Maestro, Vittorio Gallese

**Affiliations:** ^1^Unit of Neuroscience, Department of Medicine and Surgery, University of Parma, Parma, Italy; ^2^Eating Disorders Unit, Casa di Cura, “Villa Margherita”, Arcugnano, Vicenza, Italy; ^3^Istituto di Ricovero e Cura a Carattere Scientifico (IRCCS), Fondazione Stella Maris, Pisa, Italy; ^4^“GNOSIS” Research and Psychotherapy Group, Cuneo, Italy; ^5^Comunità ad alta Intensità di Cura per Minori con DNA “Gli Orti di Ada”, Calambrone, Italy; ^6^Italian Academy for Advanced Studies in America, Columbia University, New York, NY, United States

**Keywords:** anorexia nervosa, body size overestimation, body image distortion, body schema distortion, bodily self-disorders, hand laterality judgment, eating disorders, self-advantage

## Abstract

It is widely known that among others, a pervasive symptom characterizing anorexia nervosa (AN) concerns body image overestimation, which largely contributes to the onset and maintenance of eating disorders. In the present study, we investigated the nature of the body image distortion by recording accuracy and reaction times in both a group of healthy controls and AN patients during two validated tasks requiring an implicit or explicit recognition of self/other hand stimuli, in which the perceived size of the stimuli was manipulated. Our results showed that (1) the perceived size of hand stimuli modulated both the implicit and explicit processing of body parts in both groups; (2) the implicit self-advantage emerged in both groups, but the bodily self, at an explicit level (perceptual, psycho-affective, cognitive) together with the integration and the distinction between self and other, was altered only in restrictive anorexia patients. Although further investigations will be necessary, these findings shed new light on the relationship between the different layers of self-experience and bodily self-disorders.

## 1. Introduction

Body size overestimation is one of the most studied controversial clinical symptoms of anorexia nervosa (AN), and it contributes extensively to the onset, maintenance, and relapse of this disorder (Glashouwer et al., 2019). It is widely accepted that the overestimation of one's own body parts reflects a distortion of body representation leading to disturbances in the way that one's body weight or shape is experienced. Such an experience involves a multidimensional pattern, including cognitive and affective components, perception, and behaviors (Cash and Deagle, 1997; Gaudio and Quattrocchi, 2012; see Gardner and Brown, 2014; Esposito et al., 2018, for a review). While we have a large body of literature studying the latter (body image-associated processes), only a small amount of studies, to date, investigated implicit and motor mechanisms (body schema-associated processes) related to bodily self-recognition and body size estimation in both health and psychopathology. The existence of a motor experience-based representation of the bodily self that presupposes the ability to perceive and identify human bodies and in particular one's own body has been demonstrated. Such experience of self is conceptualized as the pre-reflective and the most basic level of the bodily self and corresponds to the feeling of inhabiting one's body (Gallese and Sinigaglia, 2010). It suggests that the body is primarily given to us as a “source” or “power” for action, meaning that it encompasses a variety of motor potentialities that define the scope of our interactions with the world we inhabit.

A recent study by Brown et al. (2021) showed that AN patients were significantly more likely to overestimate their body parts' size compared with healthy controls both when sensory information was provided and not. Such an overestimation was positively correlated with body dissatisfaction and drive for thinness of patients. At the same time, a large body of literature suggests that the body overestimation bias found in AN could reflect an abnormal neural processing of the body schema which disturbs the metrics of body size stored in body representation as the body in action (i.e., the so-called body schema; Schwoebel and Coslett, 2005).

This claim is supported by different studies showing that while healthy individuals are easily able to move in space without bumping into obstacles or knocking things because they can quickly recalibrate affordance perception when faced with changes in bodily dimensions (e.g., due to wearing a backpack, holding a long rod, or sitting in a wheelchair; see, e.g., Yasuda et al., 2014; Franchak, 2017; Franchak and Somoano, 2018), AN patients seem to be deficient in such a competence. Accordingly, in a motor imagery study (Guardia et al., 2010) in which participants imagined walking through a projected aperture, AN patients indicated they would rotate their shoulders for relatively larger apertures than healthy controls. It should be noted that participants did not actually perform the action of walking in this study, and it cannot be determined whether AN patients made a conscious decision about having to rotate their shoulders or not. At a more implicit level (the instructions did not disclose the true nature of the task to ensure that participants' responses were driven by automatic processes rather than conscious deliberation or strategic behavior), Keizer et al. (2013) compared AN patients' and healthy controls' performances in a body-scaled action task. Participants were asked to walk through door-like openings varying in width while performing a diversion task. AN patients started rotating for openings 40% wider than their own shoulders, while HC started rotating for apertures only 25% wider than their shoulders.

These studies suggest that, in clinical samples of AN patients, there is a relationship between body size overestimation and alterations in bodily representation associated with the body in action, but no study, to date, has directly related these two components while also taking into account both possible alterations in body schema and body image.

With the aim of addressing this research gap, the present study involved a sample of AN patients in a duly adapted version of the Implicit and Explicit Self-Other body parts recognition task (Urgesi et al., 2006, 2011; Ferri et al., 2011, 2012; Campione et al., 2017; Ardizzi et al., 2020). This widely used protocol is designed to assess and differentiate bodily sensorimotor mechanisms (associated with body schema) from perceptual mechanisms (associated with body image) related to Self-Other body parts recognition. The protocol consists of two tasks: the Implicit Self-Other body parts recognition task and the Explicit Self-Other body parts recognition task. In both tasks, participants were presented with images of the dorsal view of both right and left hands, re-oriented into various rotated positions. In the Explicit Self-Other body parts recognition task, participants were asked to judge whether the hands displayed on the screen belong to themselves or someone else (explicit self-body recognition). In contrast, in the Implicit Self-Other body parts recognition task, participants were not explicitly required to judge whether the rotated hand presented on the screen belongs to themselves or someone else (implicit self-body recognition), but they were asked to determine whether it is a right hand or a left hand. We chose this protocol because previous studies have demonstrated that when healthy participants performed the Implicit Self-Other body parts recognition task, which is a rotated hand laterality judgment task, they exhibited better or faster performance when the stimuli represented their own dominant hand rather than someone else's hand (self-advantage effect). However, when participants were subjected to the explicit self-other body parts recognition task (where explicit self-recognition was required), the self-advantage effect was absent. This dissociation highlights the involvement of different processes related to body schema and body image. Only implicit recognition of the bodily self, mapped in motor terms, allows the self-advantage to emerge (Ferri et al., 2011).

The self-advantage effect (which, as mentioned above, emerges only in the implicit self/other body part recognition task) is supported by mental motor rotation processing, which was found to be easier and faster when participants mentally rotated the images of their own body parts compared with unfamiliar ones. It is well known, indeed, that to perform the implicit task, participants simulate a mental motor rotation of their own body parts to match that of the observed stimulus (Parsons, 1994; Ionta et al., 2007), which shares the same temporal and kinematic properties with actual body rotation in space (Decety et al., 1991; Parsons, 1994; Porro et al., 1996; Jeannerod and Pacherie, 2004). A neuroimaging study, adopting this paradigm, confirmed this claim demonstrating that the previously mentioned implicit and pre-reflective sense of being a bodily self is embedded within the sensory-motor system and revealed a neural network for a general representation of the bodily self, encompassing the SMA and pre-SMA, the contralateral premotor cortex, the anterior insula, and the occipital cortex bilaterally (Ferri et al., 2012). In this case, participants used visual cues instead of covert motor processing to complete the task, thus tapping into their body image rather than body schema (Candini et al., 2016).

In the present adapted version of the Implicit and Explicit Self-Other body parts recognition task, stimuli could be presented in both their original size and fattened or slimmed down. This adjustment allows us to investigate the phenomenon of body parts overestimation in relation to patients' potential deficits in body schema or body image, eliciting the implicit and explicit body parts recognition, respectively. Both healthy participants (HCg) and restrictive anorexia (ANg) patients were submitted to the experiment with either self or others' hands as stimuli.

### 1.1. Expected results

As a result, we expected a size modulation of both implicit and explicit tasks in both HCg and ANg. We hypothesized a dissociation between implicit and explicit bodily self-recognition, which was previously found in healthy participants by Ferri et al. (2011, 2012). Additionally, we anticipated a weaker or absent implicit self-advantage in ANg compared with HCg, indicating less involvement of motor mental rotation processes during the implicit recognition of body parts. Coherently with anorexia supposed deficit in body image processing, we anticipated reduced self/other discrimination among ANg compared with HCg in the explicit experiment.

## 2. Materials and methods

### 2.1. Participants

To determine the required minimum sample size, an a priori power analysis (G^*^Power 3.1.9.7; Faul et al., 2007) was conducted for repeated-measures ANOVA considering both within and between interactions (1–ß = 0.95, α = 0.05, and effect size f = 0.25). The results indicated a required sample size of 47 participants. Thus, the obtained sample size of *N* = 52 was adequate to test our study hypothesis.

Twenty-five right-handed women with a diagnosis of restrictive anorexia nervosa (ANg; mean age: 23 years, SE = 1.9; mean BMI: 16.1 Kg/m^2^, SE = 0.3) restrictive subtype according to the DSM-IV criteria (American Psychiatric Association, 1994) and 27 healthy right-handed women (HCg; mean age: 23 years, SE = 1.1; mean BMI = 21.5 Kg/m^2^, SE = 0.6) were included in the study. They were recruited among patients hospitalized at Villa Margherita Clinic, Arcugnano, Vicenza, Italy. The control participants were recruited among voluntary students at the University of Parma by means of flyers posted at public locations and on social media. The restrictive subtype of AN is characterized by the absence, during the last 3 months, of recurrent episodes of binge eating or purging behaviors, such as self-induced vomiting or the misuse of laxatives, diuretics, or enemas. All participants were right-handed as assessed by the Edinburgh Handedness Inventory (Oldfield, 1971). Recruitment was interrupted once the two groups (matching the inclusion criteria), balanced by sex, age, and number of participants, were reached. The study enrolled anorexic patients with a BMI of at least 16. The experiment was conducted at a minimum of 1 month after their admission, or after they were able to follow the proposed food scheme for 10 days. This was done to allow for recovery from malnutrition, which could affect the performance, and to establish a minimal diet, as well as to protect the integrity of their treatment process. The patients were undergoing a multidisciplinary rehabilitation program as inpatients and thus had a pre-established meal plan consisting of breakfast, lunch, dinner, and three snacks, which was a crucial component of their treatment regimen. The meal plan adhered to the Italian National Recommended Nutrient Intake levels (LARN), the principles of the Mediterranean diet, and provided 1,900 Kcal with a composition of 17% protein, 56% carbohydrates, and 27% lipids.

Exclusion criteria for both groups included actual or past cognitive disorders (intellectual disability), psychiatric disorders (psychosis), severe medical illnesses (head trauma, neurological and cardio-respiratory diseases, and diabetes), and substance dependence. A further exclusion criterion for the control group was a personal history of eating disorders, the risk to develop eating disorders, assessed by means the Eating Disorder Inventory—EDI-3 (in particular Drive for Thinness, Body Dissatisfaction, and Eating Disorder Risk and Concern subscales; Giannini et al., 2008), the Eating Disorder Examination Questionnaire—EDE-Q (Fairburn and Beglin, 2008), which measures eating disorder behaviors and attitudes, concerns about body image and body dimension: the Body Shape Questionnaire—BSQ (Stefanile et al., 2009). Additionally, we administered the SCL-90 (Derogatis and Cleary, 1977) to exclude psychopathological symptoms in HCg (a total score higher than 1 represented an exclusion criterion from the study). Given the frequent comorbidity in ANg with major depression or personality disorders, these were not exclusion criteria for ANg. All participants provided written informed consent to participate in the study, which was approved in advance by the Clinical Center of Casa di Cura Villa Margherita, Arcugnano, Vicenza, Italy. The experiment was conducted in accordance with the ethical standards outlined in the 2013 Declaration of Helsinki.

### 2.2. Assessment

In a previous session before the experiment, participants filled in several questionnaires. Present and past participants' health history was screened thanks to an anamnestic questionnaire. To assess both the risk and symptomatology associated with eating disorders, we administered the Eating Disorder Inventory (EDI-3; Giannini et al., 2008), which consists of 91 items with a range of scores from 0 to 30 organized in 12 subscales, including drive for thinness, bulimia, perfectionism, body dissatisfaction, and interoceptive deficits, among others; the Eating Disorder Examination Questionnaire (EDE-Q; Fairburn and Beglin, 2008), which consists of 28 items with a range of scores from 0 to 6 grouped into four subscales: restraint, eating concern, shape concern, and weight concern; and the Body Shape Questionnaire (BSQ; Stefanile et al., 2009), consisting of 34 items rated from a six-point scale. To measure concerns about body shape, we used the Body Uneasiness Test (BUT; Cuzzolaro et al., 2006), composed of 71 items with a range of scores from 0 to 3, and assesses seven subscales, including body image, appearance checking, and avoidance.

To assess participants' current psychological status and to exclude psychopathological symptoms in HC, the Symptom Checklist-90 (SCL-90; Derogatis and Cleary, 1977) was administered. The SCL-90 is composed of 90 items and provides a global severity index as well as subscale scores for different psychological symptoms including somatization, obsessive-compulsive behaviors, psychoticism, and anxiety.

Lastly, to explore participants' possible dissociative symptoms, including depersonalization, derealization, and dissociative amnesia, they also filled in the Dissociative Experiences Scale (DES; Carlson and Putnam, 1993), which consists of 28 items, grouped into three subscales (amnesia, depersonalization/derealization, and absorption) with a range of scores from 0 to 100.

During the experimental session, to assess depressive and anxious states, participants completed Beck's Depression Inventory II (Beck et al., 1996; Ghisi et al., 2006; BDI-II) and the State-Trait Anxiety Inventory (Pedrabissi and Santinello, 1989). Sociodemographic features, pharmacological data, and questionnaire scores obtained from the two groups of participants are shown in Tables 1, 2.

**Table 1:** Comparison between the two groups with respect to socio-demographic and questionnaire data (n.a. = not applicable).

	**AN mean (SE)**	**HC mean (SE)**	**T (df = 1,48)**	***p***
N (sex)	25 (f)	27 (f)	n.a.	n.a.
Age	23 (1.9)	22.7 (1)	−0.07	0.87
Age of onset	16.7 (0.6)		n.a.	n.a.
Years of illness	6.3 (1.6)		n.a.	n.a.
BMI	16.1 (0.3)	21.5 (0.6)	8.11	<0.001
BSQ	121.3 (8.3)	69 (4.5)	−5.71	<0.001
SCL-90—Global Symptomatic Index	1.4 (0.1)	0.5 (1.3)	−5.61	<0.001
DES	23.6 (3.5)	7.5 (1.4)	−4.21	<0.001
STAI Trait	62.7 (2)	41.1 (1.6)	−8.5	<0.001
STAI State	49.8 (2.2)	36 (1.9)	−4.8	<0.001
BDI	27.2 (2.7)	8.9 (1.8)	−5.71	<0.001
EDI-3—Drive For Thinness	72.5 (6.2)	27.2 (5.5)	−5.4	<0.001
EDI-3—Body Dissatisfaction	75.2 (3.9)	42.3 (5)	−5	<0.001
EDI-3—Emotion Dysregulation	65 (5.5)	32.5 (4.9)	−4.4	<0.001
EDI-3—Eating Disorder Risk Composite	74.2 (3.9)	36.5 (4.3)	−6.3	<0.001
EDI-3—Interoceptive Deficits	77.5 (5.2)	32.5 (5.5)	−5.9	<0.001
EDI—Bulimia	49 (6.9)	34.1 (4.6)	−1.81	0.07
EDI-3—Low Self Esteem	81.8 (4.2)	39.2 (5.6)	−6	<0.001
EDI-3—Global Psychological Maladjustment Composite	78.9 (5.1)	37.5 (5.3)	−5.6	<0.001
EDI-3—Interpersonal Problem Composite	72.2 (5.1)	44.2 (5.5)	−3.7	<0.001
EDE-Q—Global score	55.7 (6.8)	3.9 (1.6)	−7.41	<0.001
EDE-Q—Restraint	10.3 (1.4)	2.6 (1.3)	−3.9	<0.001
EDE-Q—Eating concern	11.3 (1.3)	1.2 (0.9)	−6.11	<0.001
EDE-Q—Weight concern	16.6 (1.7)	2.3 (1.1)	−7.21	<0.001
EDE-Q—Shape concern	34 (2.6)	2.8 (1.2)	−10.81	<0.001
BUT—Global Severity index	2 (0.1)	1 (0.1)	−3.91	<0.001

**Table 2:** Number of participants taking psychiatric medications at the time of the experiment.

	**ANg (N)**	**HCg (N)**
Psychiatric drug assumption	13/25	0/27

### 2.3. Stimuli and procedure

**Figure 1:**
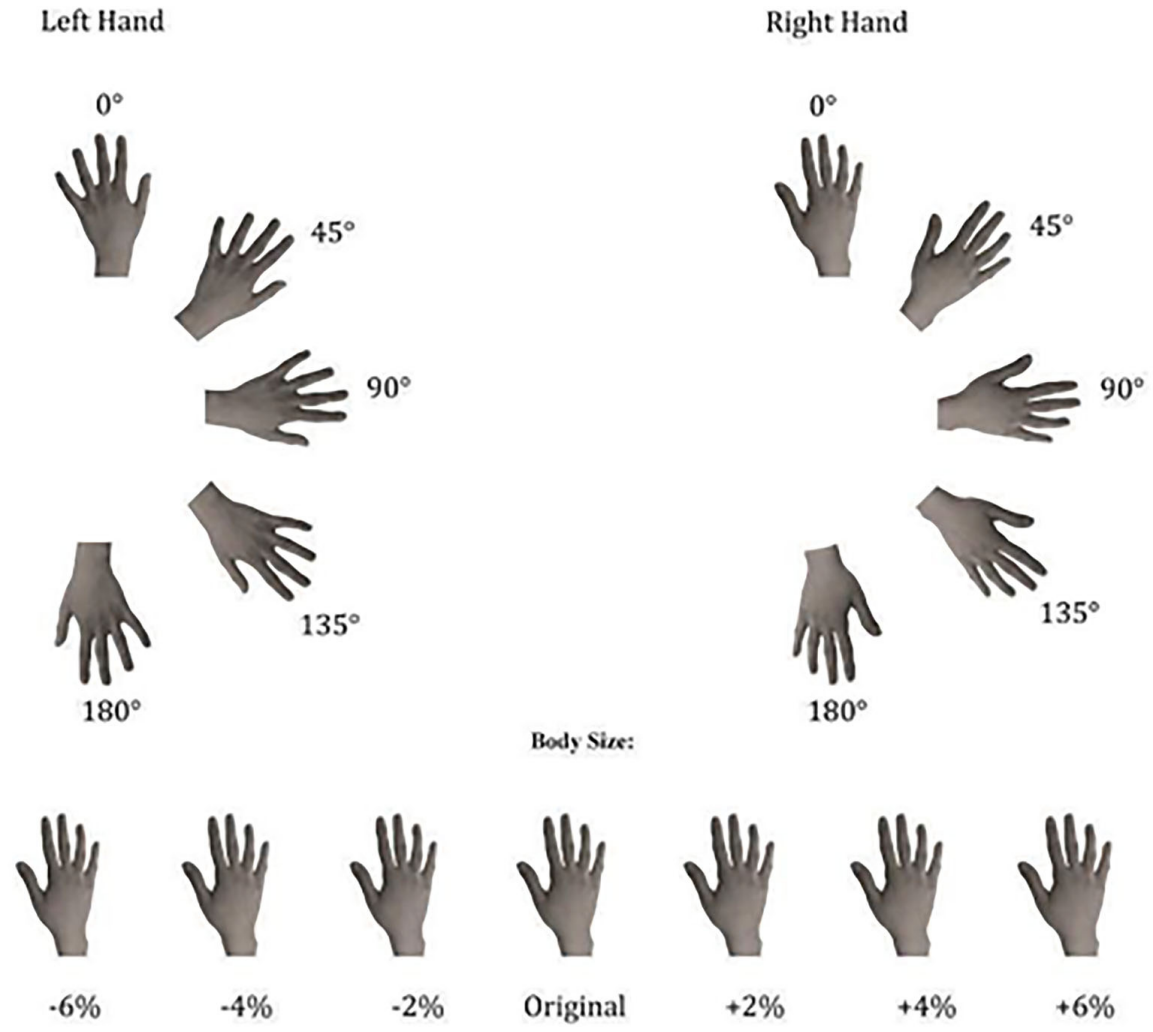
Experimental stimuli used in both implicit and explicit experiments, depicting the dorsal view of right and left hands in seven different sizes and five different clockwise orientations.

The experimental stimuli consisted of grayscale pictures of the dorsal view of participants' right and left hands. In the session prior to the experiments (at least 1 week before the experimental session), the hands of each participant were photographed with a digital camera. This session took place in a controlled environment with constant artificial light and a fixed distance from the camera lens to participants' hands (40 cm) photographed always in the same position. Then, with Adobe Photoshop software, photographs were cut from the original picture and modified in their horizontal dimension in order to provide a “Weight Gain” (+2%, +4%, +6% in width) or a “Weight Loss” (−2%, −4%, −6% in width) effect (see Figure 1). Subsequently, the obtained images were pasted on a white background and re-oriented into the different rotated positions (0°, 45°, 90°, 135°, and 180°). Other people's hands were selected from this database as the best match for size, skin color, and age, in comparison with each participant's hands. Hands' size was matched to minimize the differences both in length and in width of the stimuli. Each stimulus was presented, one at a time at the center of the computer screen in the five different clockwise orientations from the upright one (0°). The upright orientation was defined as fingers pointing upward (see Figure 1).

Participants sat in front of a PC screen, at a distance of about 30 cm. Stimuli presentation was controlled by E-Prime (Psychology Software Tools, Inc.). Each trial started with a central fixation cross (500 ms duration), followed by stimulus presentation. The trial was timed out as soon as participants responded (up to 4,000 ms).

**Figure 2:**
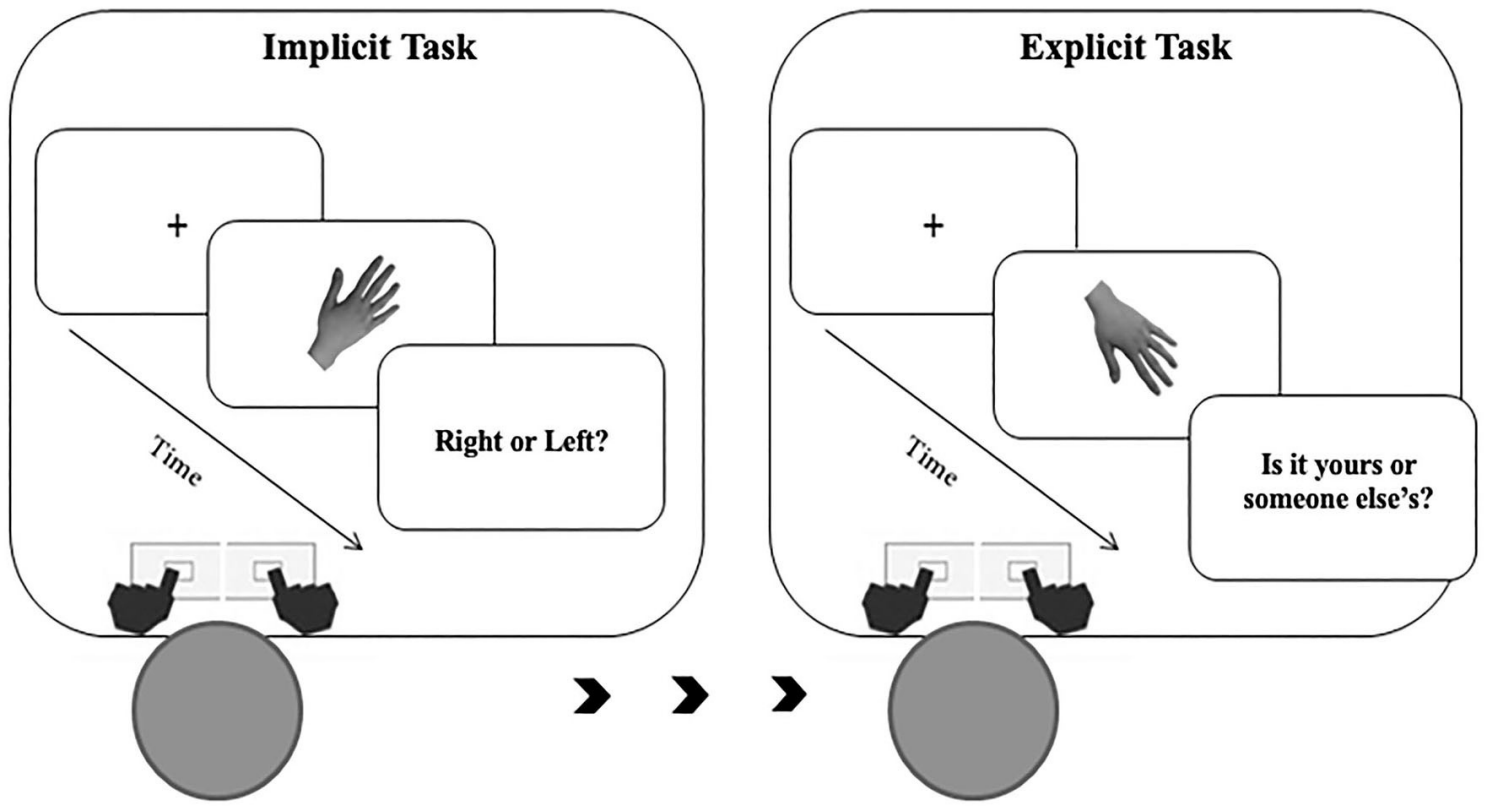
Description of the experimental paradigm and order of events.

In the implicit experiment (see Figure 2), participants were required to judge the laterality (left or right) of the observed digital images of hands by pressing as accurately as possible and within the allowed time interval, a left or a right response key, with their left and right index fingers, respectively.

In the explicit experiment (see Figure 2), participants were required to explicitly judge whether the presented stimulus represented their own hand or other's hand by pressing as accurately as possible and within the allowed time interval, a left or a right response key, with their left and right index fingers, respectively. The response keys were counterbalanced between participants.

Each experiment consisted of 420 trials. Stimuli depicted the participant's own left or right hand in half of the trials (210 self-trials). In the other half of the trials, stimuli depicted the right or left hand of the other three people (210 other trials). Each hand stimulus was presented in three different size conditions: “Weight Gain”: +2%, +4%, +6%; “Original size”: 0%; “Weight Loss” or “Thin”: −2%, −4%, −6%). This means that in the experiment, we had three levels for the variable: Thin (Weight Loss), Original (Original size), and Fat (Weight Gain). This indicates that within the Thin condition, the hand stimuli were proportionally reduced by 2, 4, and 6% of their original size. In the Original condition, the presented images remained unchanged at their original size. Lastly, in the Fat condition, the hand stimuli were proportionally increased by 2, 4, and 6% of their original size. Each weight condition was randomly presented 15 times. Tasks were always preceded by a task-specific practice block. The implicit task was always conducted before the explicit task (see Figure 2). Reaction times (RTs, times elapsed between the stimuli presentation and spacebar pressure) and accuracy rates (percentage of correct answers) were recorded.

## 3. Results

For each experiment, trials in which participants failed to respond correctly were excluded from the analysis (in the implicit experiment, 12.9% of 21,840 trials; in the explicit experiment, 17.1% of 21,840 trials). We used the Jamovi software for statistical analyses (The Jamovi Project, 2021). For both implicit and explicit tasks, participants' correct responses (accuracy arcsine transformed), response times (RTs), and slopes (that is, the measure of changes associated with motor mental rotation; Ionta et al., 2007) entered in repeated-measures ANOVA with Group (HCg vs. ANg) as a between-group factor and Owner (one's own and other people's stimuli), Laterality (left and right), and Size (Thin, Original, and Fat) as within-group factors. The slope specifically captures the aspects related to the mental rotation process, representing the average change associated with each additional degree of object rotation. In our study, we employed the methodology outlined by Ionta et al. (2007) to calculate the slopes. This involved conducting regression analyses on the response times (Rts) for various stimulus orientations and extracting the slope values. For each analysis, we conducted a series of preliminary tests to verify assumptions, including the Shapiro–Wilk test to assess normality and Mauchly's test for homogeneity of variances. In case of any violations of these assumptions, we reported it in the manuscript describing the applied non-parametric tests in case of normality violation and the appropriate correction, such as the Greenhouse–Geisser correction for sphericity and the Welch correction for homogeneity of variances. Finally, we conducted *post hoc* tests to investigate significant main effects and interactions, using the Holm correction for multiple comparisons.

In the explicit experiment, to be sure that responses were the product of the self/other discrimination rather than chance (signal detection theory, Pastore and Scheirer, 1974), we computed a signal detection analysis able to estimate explicit self/other discrimination, given the incidence of potential response and we compared the D Prime of both group by means an independent-sample *t*-test.

### 3.1. Implicit task

#### 3.1.1. Accuracy

In line with our hypothesis, the main effect of Size was significant [F_(2, 100)_ = 7.88; *p* < 0.002; = 0.14; Greenhouse-Geisser corrected], with a significantly higher percentage of correct responses for the Original size (89%; SE = 0.03) than both Thin (88%; SE = 0.02) (*p* < 0.01) and Fat sizes (88%; SE = 0.01) (*p* < 0.01).

#### 3.1.2. Reaction times

The analysis showed the significance of Group [F_(1,50)_ = 5.99; *p* < 0.05; = 0.11] with slower RTs for ANg (EMM = 1,323 ms, SE = 64.8) than HCg (EMM = 1,099 ms, SE = 64.8).

Coherently with the experimental effect, the main effect of Laterality was also significant: [F_1, 50)_ = 21.103; *p* < 0.001; = 0.30], demonstrating for both groups better performances for stimuli depicting right (dominant) (EMM = 1,169 ms, SE = 46.7) than left hands (EMM = 1,253 ms, SE = 46.7).

Even the main effect of Size was significant [F_(2, 100)_ = 3.47; *p* < 0.03; = 0.07], with slower RTs in the Fat (EMM = 1,227 ms; SE = 46.3) than both Thin (EMM = 1,206 ms; SE = 46.3) (*p* < 0.05) and Original conditions (EMM = 1,199 ms; SE = 46.3) (*p* < 0.01).

**Figure 3:**
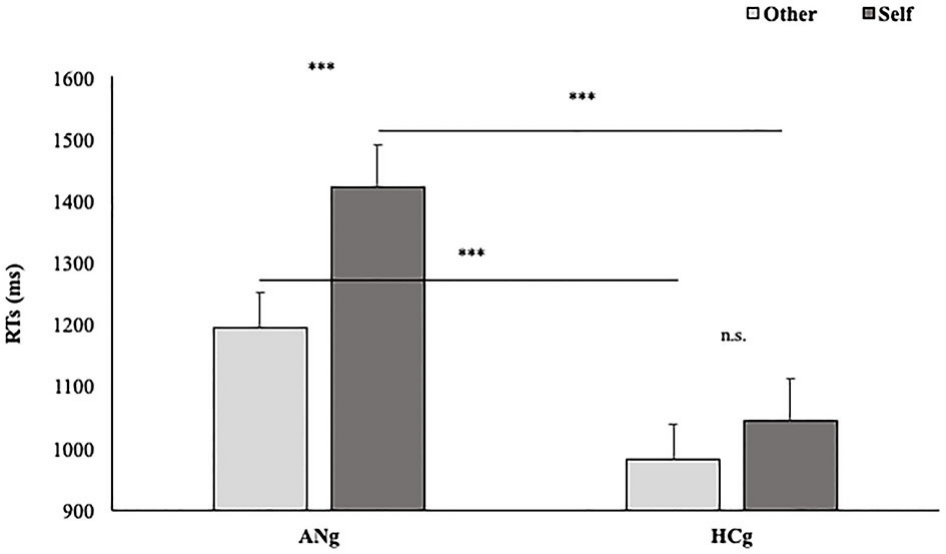
Estimated marginal means of participants' RTs in the function of Groups (HCg vs. ANg) and Laterality (R = Right vs. L = Left). Error bars depict the standard error of the mean. ***p < 0.001; n.s., not significant.

Coherently with our hypothesis, the interaction between Group and Laterality was significant [F_(1,50)_ = 5.9; *p* < 0.05; = 0.11], showing, only for HCg, faster RTs for trials depicting right (EMM = 1,304 ms; SE = 66.1) than left hands (EMM = 1,163 ms; SE = 66.1) (*p* < 0.001). Such a difference did not emerge in ANg (Right: EMM = 1,303 ms; SE = 66.2 vs. Left: EMM 1,343 ms; SE = 66.2; *p* > 0.27); HCg was significantly faster than ANg (*p* < 0.05) responding to stimuli depicting right hands (see Figure 3).

The interaction between Ownership and Laterality was significant [F_(1,50)_ = 4.2; *p* < 0.05; = 0.10], with faster RTs in the Self (EMM = 1,148 ms; SE = 47.5) than the Other condition (EMM = 1,189 ms; SE = 47.5) (*p* = <0.05) only for Right stimuli (i.e., the self**-**advantage), but, contrarily to our hypothesis, not only in HCg but for both groups. Conversely, for stimuli depicting left hands, the difference between Self and Other conditions was not significant (Self: EMM = 1,251 ms; SE = 47.5; Other: EMM = 1,255 ms; SE = 47.5).

The interaction among Group, Ownership, and Laterality was not significant [F_(1,50)_ = 0.35; *p* > 0.5; = 0.01]; therefore, the performances of both HCg and ANg seem not to differ in their self-advantage for the right stimuli.

#### 3.1.3. Pharmacological treatment

To test whether the overall slowness of RTs of ANg compared with HCg was due to the influence of pharmacological treatment rather than the stimuli processing speed, two independent-sample *t*-tests comparing patients taking medications (PTM) with patients not under medication (PNM) and HCg with PNM were carried out. They showed significantly slower performances for PTM (EMM = 1,480 ms; SE = 130) than PNM (EMM = 1,162 ms; SE = 67.4) (T23 = 2.13; *p* < 0.05; Cohen's *d* = 0.85 Welch corrected; Cohen, 1969). Coherently, the performance PNM (EMM = 1,162 ms; SE = 67.4) resulted in no significant difference from the HCg (EMM = 1,103 ms; SE = 47.7) (T37 = 0.7; p = 0.5; Cohen's *d* = 0.24).

Slopes: The ANOVA showed the main effect of Size [F_2, 76_ = 19; *p* < 0.001; = 0.33], resulting in flattened slopes for the Original size (0.14; SE = 0.01) than both Thin (0.20; SE = 0.01) (*p* < 0.001) and Fat (0.22; SE = 0.01) sizes (*p* < 0.001).

As expected, the main effect of Laterality was significant [F_1, 38_ = 19; *p* < 0.05; = 0.14], showing significantly higher slopes for stimuli representing right (0.199; SE = 0.01) than left hands (0.181; SE = 0.01).

**Figure 4:**
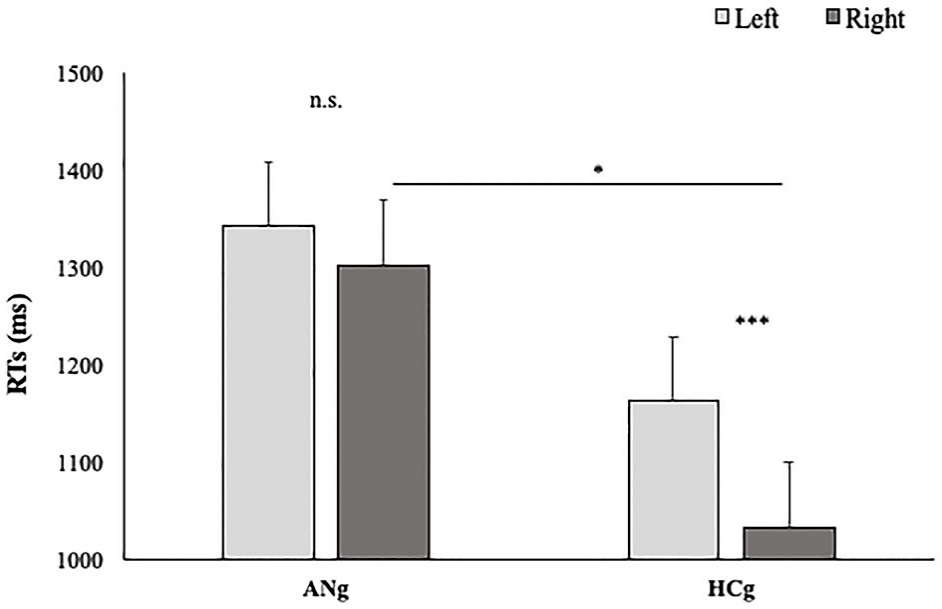
Slopes of participants' RTs in function of Groups (HCg vs. ANg) and Laterality (Right vs. Left). Error bars depict the standard error of the mean. n.s. = not significant; *P < 0.05; ^***^p < 0.001; n.s., not significant.

The factor Group interacted significantly with the factor Laterality [F_(1,38)_ = 12.1; *p* < 0.01; = 0.25], showing higher slopes in the Right than (0.22; SE = 0.02) Left condition (0.18; SE = 0.02) (*p* < 0.001) in HCg. Such a difference, interestingly, was not significant in ANg (Left: 0.18; SE = 0.02; Right: 0.17; SE = 0.02) (*p* > 0.9) (see Figure 4).

### 3.2. Explicit task

#### 3.2.1. Accuracy

The main effect of Group was significant [F_(1,46)_ = 12.9; *p* < 0.001; = 0.22] showing a lower accuracy in ANg (ANg = 75%, SE = 0.03; HCg = 91%, SE = 0.04).

**Figure 5:**
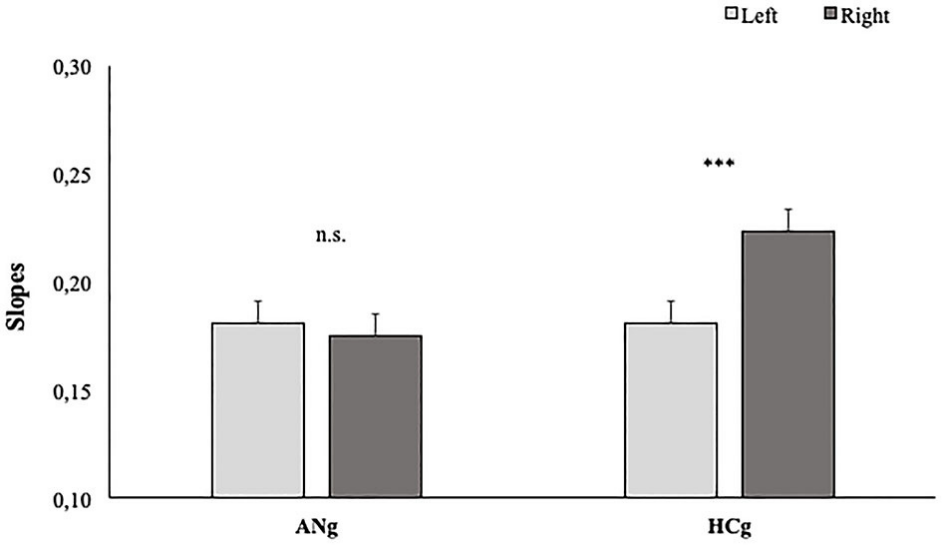
Mean percentage of participants' accuracy in function of Size. Error bars depict the standard error of the mean. ^***^p < 0.001; n.s., not significant.

In line with our hypothesis, the main effect size was also significant [F_(2, 92)_ = 5; *p* < 0.01; = 0.10]. Participants gave more accurate responses in the Fat (84.5%, SE = 0.02) than Thin (82.2%; DE = 0.02) condition (*p* < 0.01) (see Figure 5).

The interaction between Size and Ownership was significant [F_(2, 92)_ = 4.1; *p* = <0.05; = 0.08] with higher performances for stimuli depicting other's hands in the Fat (85%, SE = 0.03) than Thin (81.6%, SE = 0.03) condition. Even the interaction between Size and Laterality was significant [F_(2, 92)_ = 5.2; *p* < 0.01; = 0.10] resulting in higher accuracy for thinner stimuli depicting left (83.4%, SE = 0.03) than right hands (81%, SE = 0.03) (*p* < 0.01). Participants were also more accurate when the right stimuli were presented in a fatter than thinner size (Fat: 84.6%, SE = 0.03; Thin: 81%, SE = 0.03) (*p* < 0.001).

Finally, the factors Size, Ownership, and Laterality interacted significantly [F_(2, 92)_ = 5.2; *p* < 0.01; η^2^ = 0.10], indicating better performance when the stimuli presented were the right hands of others in a fatter size rather than a thinner size (Fat: 85%, SE = 0.03; Thin: 80%, SE = 0.03) (*p* < 0.01).

#### 3.2.2. Pharmacological treatment

As previously mentioned, to assess the possible influence of medication on the main effect of Group, an independent-sample *t*-test was conducted comparing RTs of PTM with those of PNM. The analysis did not show any significant difference (PTM = 70%; SE = 0.09; PNM = 80%; SE = 0.03) (T23 = 0.16; *p* > 0.8 Cohen's *d* = 0.07).

### 3.3. Reaction times

The main effect of the Group was significant [F_(1,46)_ = 13.6; *p* < 0.001; = 0.23] with HCg showing Faster RTs (EMM = 1,006 ms, SE = 57) compared with ANg (EMM = 1,304, SE = 57).

In line with our hypothesis, also the effect of Size was significant [F_(2, 92)_ = 4.77; *p* = 0.01; = 0.10] showing faster RTs when stimuli were presented in the Original (EMM = 1,137 ms, SE = 40.7) than Fat size (EMM = 1,169 ms, SE = 40.7) (*p* = 0.009). The thin (EMM = 1,158 ms, SE = 40.7) condition did not significantly differ from other conditions (*p* > 0.05).

Reaction Times. The main effect of Ownership was also significant [F_(1,46)_ = 25.5; *p* < 0.001; = 0.36] showing better performances in both groups for Other than Self Trials (Self: EMM = 1,227 ms, SE = 42.7; Other: EMM = 1,083 ms, SE = 42.7). The interaction between Ownership and Size was also significant [F_(2, 92)_ = 3.2; p = 0.04; = 0.10]. *Post hoc* comparisons showed faster RTs for other's trials presented in the original size (EMM = 1,059, SE = 43.8) than for thinner size (EMM = 1,105, SE = 43.8) (*p* = 0.03).

**Figure 6:**
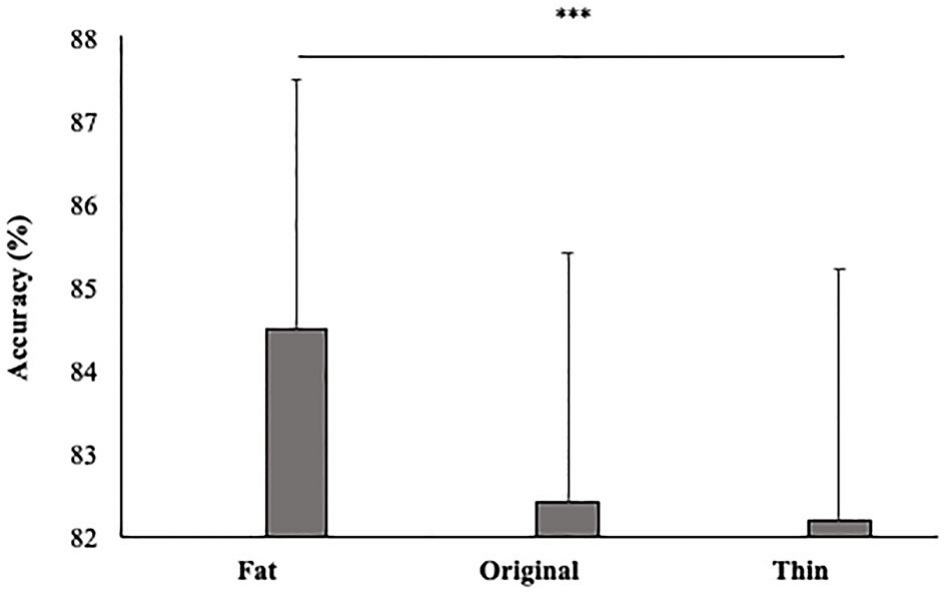
Estimated Marginal Means of participants' RTs in function of Group (AHg vs. HCg) and Ownership (Other vs. Self). Error bars depict the standard error of the mean. ^***^p < 0.001.

The effect of Group interacted significantly with Ownership [F_(1,46)_ = 8.5; *p* < 0.05; = 0.16] with faster performances among ANg in response to Other (EMM = 1,190 ms, SE = 60.6) than Self (EMM = 1,417 ms; SE = 60.6) stimuli (*p* < 0.001). Conversely, no significant differences were found between self and other stimuli in HCg (Other: EMM = 976 ms, SE = 60.3; Self: EMM = 1,037 ms, SE = 60.3) (*p* > 0.1). Moreover, ANg showed significantly slower RTs than HCg for both Self (*p* < 0.001) and Other stimuli (*p* < 0.001) (Figure 6). Therefore, in line with the experimental effect, no self-advantage emerged.

The factors, Ownership, Laterality, and Size, interacted significantly [F_(2, 92)_ = 3.4; *p* < 0.05; = 0.10]. Concerning right others' stimuli, *post hoc* comparisons did not detect any significant difference in RTs along the three sizes (Fat: EMM = 1,103 ms; SE = 46.8; Original: EMM = 1,080 ms, SE = 46.8; Thin: EMM = 1,091, SE = 46.8; *p* > 0.05). On the contrary, concerning left others' stimuli, participants showed slower RTs for thinner than Original and fatter stimuli (Fat: EMM = 1,066 ms; SE = 46.8; Original: EMM = 1,037 ms, SE = 46.8; Thin: EMM = 1,120 ms, SE = 46.8) (*p* < 0.001). Regarding self-stimuli, both for right and left hands, *post hoc* comparisons did not show significant differences along the three sizes [Self Left (Fat: EMM = 1,268 ms, SE = 46.8; Original: EMM = 1,229 ms, ES = 46.8; Thin: EMM = 1,212 ms, ES = 46.8); Self-Right (Fat: EMM = 1,238 ms; SE = 46.8; Original: EMM = 1,203 ms, SE = 46.8; Thin: EMM = 1,211 ms; SE = 46.8) (all ps > 0.4)].

Regarding others' stimuli, when they are presented Fatter and right, they led to faster RTs when stimuli belonged to Others (EMM = 1,103 ms, SE = 46.8) than Self (EMM = 1,238 ms, SE = 46.8) (*p* < 0.05), and to faster RTs when the presented stimuli were left (EMM = 1,066 ms, SE = 46.8) than right hands (EMM = 1,268 ms, SE = 46.8) (*p* < 0.001). Finally, for left stimuli presented in the original size, participants showed faster performances responding to others (EMM = 1,037 ms, SE = 46.8) than self-stimuli (EMM = 1,229 ms, SE = 46.8) (*p* < 0.05).

Pharmacological treatment: To assess whether the overall slowness of RTs of AN compared with HC was due to the influence of pharmacological treatment rather than the processing of the stimuli, an independent-sample *t*-test was conducted comparing Rts of PTM with those of PNM. The analysis did not show any significant difference between PTM (EMM = 1,279 ms; SE = 89.5) and PNM (EMM = 1,270 ms; SE = 72.6) (T23 = 0.7; *p* > 0.9 Cohen's *d* = 0.03).

### 3.4. D prime

**Figure 7:**
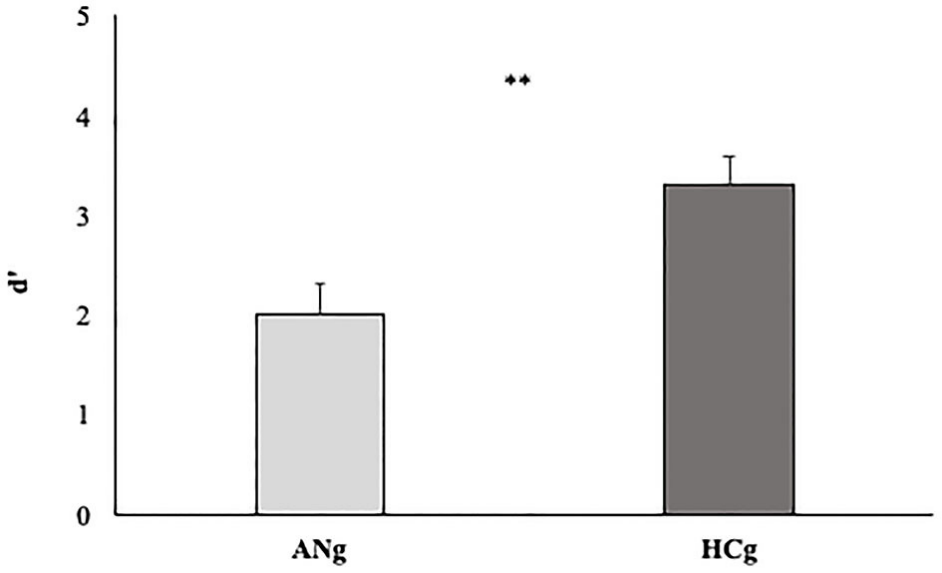
D' prime rates of the two groups (ANg and HCg). Error bars depict the standard error of the mean. ^**^p < 0.01.

To test the D prime of the two groups, an independent-sample *t*-test was carried out. A significant difference between HCg and ANg emerged (T_48_ = 3.24 *p* < 0.0; Cohen's *d* = 0.92 Welch corrected) highlighting higher d prime rates for HCg (3.3, SE = 0.2) compared with ANg (2, SE = 0.3) (see Figure 7).

This result aligns with our initial hypothesis.

## 4. Discussion

The current study investigated whether, and at which level, the overestimation of own body size, typical of people affected by restrictive AN, could affect bodily self-recognition, taking into account both pre-reflective (the motor representation of the bodily, self-influencing their implicit self-advantage) and perceptual cognitive and affective (i.e., the body image) levels of bodily self-processing. Furthermore, it also aimed to assess how the manipulation of the perceived size of hand stimuli could modulate both explicit and implicit self-recognition in both HCg and ANg. Participants were submitted to an adapted version of the implicit and Explicit Self-Other body parts recognition task by Ferri et al. (2011).

The results of the Implicit task corroborated previous findings showing better performances (in terms of accuracy and response velocity) for right than for left hand stimuli, confirming once again that right-handed participants took advantage of a pragmatic motor hand representation only when a laterality judgment was required (Gentilucci et al., 1998; Ferri et al., 2011; Ardizzi et al., 2020). The involvement of motor mental rotation processes during this task is confirmed by higher slope values for right than left stimuli. Importantly, this latter result emerged only in HCg, and sure enough, they showed not only significantly faster performance responding to right than left stimuli compared with ANg but also faster performance when responding to right stimuli than ANg. In addition, as HCg showed also higher slope values for right than left stimuli, and given that such dissociation was not present in ANg, these results suggest that mental motor processing is more involved in HCg than ANg during the performance. This result is in line with Scarpina et al. (2022) and Meregalli et al. (2023) showing that in a hand laterality Judgment Task, anorexia patients showed altered imagery processes not adopting motor strategies, as shown by the absence of biomechanical constraint effect (i.e., faster and more accurate responses for medial than lateral rotations, and back than palm view for palm down posture). It is interesting to note that in a recent study by Ardizzi et al. (2020) involving a group of schizophrenia patients in the Implicit Self-Other body parts recognition task, the authors found a specific deficit in patients' mental rotation (i.e., low slope values) of self-body parts resulting in the absence of the expected Self-advantage effect. Even if schizophrenia and anorexia represent two distinct disorders and lead to a specific symptomatology, they share some common alterations in living their bodily self, resulting in an altered integration of multisensory bodily inputs (exteroceptive and interoceptive). The latter has been proposed as the basis of the non-conceptual and pre-reflective representation of the bodily self (e.g., Gallagher, 2000, p. 15; Haggard et al., 2003; Ferroni and Gallese, 2023).

Furthermore, as expected, in HCg, the dissociation between implicit and explicit bodily self-recognition found in Ferri et al. (2011, 2012) was confirmed. Participants showed the so-called self-advantage only in the implicit task, and its lack when an explicit discrimination between self and others' hands was made, leading to a sort of “other advantage.” In contrast to our hypothesis, the self-advantage occurred in both HCg and ANg demonstrating that the ownership of the stimulus aligned ANg performance to that of controls. This evidence leads to hypothesize that AN motor representation of bodily self, although altered, seems to be sufficient to facilitate the mental rotation of the preferred hands (e.g., Gallese and Sinigaglia, 2010). Such a result was also found in Campione et al. (2017) who used a similar laterality judgment task in which ANg and HCg had to mentally rotate their own and others' hands.

There are different possible explanations for this result, apparently inconsistent with studies mentioned in the introduction, showing instead a significant alteration in processes related to the body schema of anorexic patients: A first possible explanation could be that motor functions responsible for basic kinematic topographies shared by mental rotation and actual action execution are only weaker in ANg. A second possible explanation may be that, during the hand laterality judgment, compensatory strategies, such as visuospatial transformation (Kosslyn et al., 2001; Vannuscorps et al., 2012; Conson et al., 2013; Mibu et al., 2020; Scarpina et al., 2022), excessive checks on the body, and excessive concern for body image and nutrition (typical of the very nature of this disorder), are involved in ANg. A study by Bellard et al. (2021), for example, found an advantage in implicit recognition of larger body parts for (healthy) adult participants who showed high scores on body concerns (investigated through the BUT and GSI). Although our results do not allow us to strongly disambiguate between these two hypotheses, they point toward the use of compensatory strategies; in fact, although not significantly, in our study the slopes are modulated by ownership and laterality of the stimuli in HCg (in favor of self-right hands) but not in ANg. Moreover, even if ANg did not differentiate between left and right hands in the implicit task as HCg did (both in terms of RTs and Slopes), they showed differentiation in terms of RTs between other and self-stimuli (in favor of other's stimuli) in the explicit task. This result may further support the hypothesis that the underlying process of self-body recognition in terms of sensorimotor mechanisms is likely insufficient to determine the self-advantage in AN patients, but compensatory strategies of a more perceptual and cognitive nature may be involved.

Concerning the implemented change in the present protocol to observe the role of the size of body parts, results showed that even small changes in the size of the stimuli modulated the overall performance of both implicit and explicit tasks and, interestingly, in both ANg and HCg. As shown by the results of the explicit experiment, in both groups the size of the stimuli affected their performance leading to faster RTs for original size stimuli (specifically when stimuli represented left others' hands in both original and thinner sizes), but more accurate performances for fatter stimuli (specifically when stimuli were right others' hands). Additionally, in the explicit task, there is significant interaction among Size, Ownership, and Laterality for both accuracy and RTs, demonstrating that changes in body size modulate the explicit recognition (body image processes) in the function of the ownership and the laterality of the stimulus. These results are supported by findings of a large body of literature studying the body image of patients affected by eating disorders and healthy participants (e.g., Guardia et al., 2010), which highlighted a mismatch between the actual size of participants' hands and their stored (perceptual, cognitive, affective) body representation. In particular, Bellard et al. (2022) assessed whether healthy older, compared with younger women, differed in the ability of recognizing their own with respect to other women's body parts (stomach, hand, thighs, and foot) by means of an implicit task consisting of visual matching of self and others' body parts and an explicit self–other body discrimination task. Results showed that both groups were comparably less able to explicitly recognize their body parts when they appeared thinner as compared to their actual size. In addition, no difference was detected in the processing of rounder vs. actual-sized body parts.

In the implicit experiment (as emerged by the significance of the main effect “size”), participants were faster and more accurate when responding to the original size and showed more flattened slopes in this condition compared with thin and fat size. This finding is in line with Longo and Haggard (2010) who, studying the human sense of body part's position, which refers to a stored body model of the body's metric properties such as body part size and shape, developed a technique to isolate and measure this body model. Participants judged the location in the external space of 10 landmarks on the hand. By analyzing the internal configuration of the locations of these points, they produced implicit maps of the mental representation of hand size and shape of the hand. These authors discovered not only that this part of the body model was distorted, featuring shortened fingers and broadened hands, but also intriguingly, these distortions appeared to retain several characteristics of primary somatosensory representations, such as the Penfield homunculus.

In addition, also an affective involvement could be speculated to have influenced bodily self-processing, considering that our HC group included a sample of participants composed only of young female students. Devue et al. (2007), indeed, exploring brain activity during a task in which participants had to implicitly recognize their body, found that the observation of an altered version of one's own body elicited activity in prefrontal and limbic areas only in female participants, while for men it rather elicited activity in the right occipital cortex.

The last point to be addressed is that during the explicit task, ANg were slower and much less accurate (regardless of taking medications), both in the overall performance and responding to self vs. others stimuli, indicating that it appears significantly easier for individuals with anorexia nervosa to respond to stimuli related to others rather than to oneself. Furthermore, signal detection analysis showed that ANg performed worse than HCg in discriminating between self and other, suggesting that the integration and distinction between self and other in anorexia patients are not effortless, spontaneous, and balanced processes as observed in healthy controls. This impairment in self/other contributed to the complex nature of body disturbances in anorexia nervosa.

Potential limitations of this study include the small sample size and the restriction of HCg participants to students. Additionally, we did not measure participants' “body size estimation,” and it is possible that the stimuli used to assess it represented a more “neutral” body part than others, such as full-body silhouettes, the belly, or the thighs. Finally, despite the fact that it has been widely demonstrated that mental rotations of body parts place a stronger emphasis on the motor representation of the bodily self compared with mental rotations of objects (i.e., Parsons, 1994; Kosslyn et al., 1998; Wraga et al., 2005; Zacks, 2008), we did not administer any specific control tasks focusing on non-body-related processes.

## 5. Conclusion

In conclusion, our data together with previous findings highlight that (1) even small changes in body size modulate both implicit and explicit processes of body parts both in healthy controls and anorexia patients; (2) although processes associated with motor imagery of body parts seem to be stronger in healthy controls, the implicit self-advantage is preserved in anorexia patients; and (3) bodily self, at an explicit level (perceptual, psycho-affective, cognitive) together with the integration and the distinction between self and other, is altered in restrictive anorexia patients.

All in all, although further investigations will be necessary, these findings shed new light onto the relationship between the different layers of self-experience and how damage to one of these layers may lead to different symptomatology, opening up new therapeutic approaches for bodily self-disorders. The potential therapeutic approaches that may arise from these findings could involve interventions that aim to address the specific layer(s) of self-experience affected in each type of bodily self-disorder in a targeted way. For instance, if the proprioceptive-motor layer is affected, therapies aimed at improving body awareness and coordination, such as body-oriented psychotherapy, may be effective. If the disorder is associated with disturbances in body schema, exploring the effectiveness of motor imagery techniques in improving body representation and self-advantage in anorexia patients, as well as examining the potential benefits of interventions that focus on integrating and distinguishing self and other in the context of bodily self-disorders, could be beneficial. If body image is affected, cognitive-behavioral therapy may be more effective. In the case of disturbances at the affective level, interventions focused on emotion regulation and stress management, such as cognitive-behavioral therapy or mindfulness-based interventions, may be suggested. Regarding cognitive layer alterations, psychotherapeutic approaches focused on self-reflection and meaning-making, such as psychodynamic therapy or narrative therapy, may be more appropriate. However, further research is needed to determine the most effective and personalized interventions for each specific type of bodily self-disorder and to explore the generalizability of findings to other populations with body image disturbances.

## Data availability statement

The raw data supporting the conclusions of this article will be made available by the authors, without undue reservation.

## Ethics statement

The studies involving human participants were reviewed and approved by the Direzione Sanitaria della Casa Di Cura Vila Margherita represented by Dr. Giuseppe Butera. Written informed consent to participate in this study was obtained from the participants themselves, or in the case of minor participants, from their legal guardian/next of kin.

## Author contributions

MAm designed the study, collected, analyzed, interpreted the data, and wrote the manuscript. MAr involved in study design, collection of data, date analyses, and contributed to the drafting of the manuscript. ER and FD were principally engaged in the recruitment of participants and data collection, furthermore, they contributed to interpretation of results. MS, PV, PT, and SM were involved in the recruitment of participants and data collection and took part in the interpretation of the results. VG designed the study, interpreted the data, and drafted the manuscript. All the authors approved the final version of the manuscript.

## References

[B1] American Psychiatric Association. (1994). Diagnostic and Statistical Manual of Mental Disorders (4th edn.).

[B2] Ardizzi M. Ambrosecchia M. Buratta L. Ferri F. Ferroni F. Palladini B.. (2020). The motor roots of minimal self disorders in schizophrenia. Schizophrenia Res. 218, 302–303. 10.1016/j.schres.2020.03.007 32171636

[B3] Beck A. T. Steer R. A. Brown G. K. (1996). Beck Depression Inventory-II (BDI-II). San Antonio, TX: The Psychological Corporation.

[B4] Bellard A. Urgesi C. Cazzato V. (2022). Self-body recognition and attitudes towards body image in younger and older women. Arch. Women's Mental Health 25, 107–119. 34331575 10.1007/s00737-021-01164-x PMC8784361

[B5] Bellard A. M. Cornelissen P. L. Mian E. Cazzato V. (2021). The ageing body: contributing attitudinal factors towards perceptual body size estimates in younger and middle-aged women. Arch. Women's Mental Health 24, 93–105. 10.1007/s00737-020-01046-8 32562005 PMC7929965

[B6] Brown T. A. Shott M. E. Frank G. K. (2021). Body size overestimation in anorexia nervosa: contributions of cognitive, affective, tactile and visual information. Psychiatry Res. 297, 113705. 10.1016/j.psychres.2021.113705 33472094 PMC11537156

[B7] Campione G. C. Mansi G. Fumagalli A. Fumagalli B. Sottocornola S. Molteni M.. (2017). Motor-based bodily self is selectively impaired in eating disorders. PloS ONE 12, e0187342. 10.1371/journal.pone.0187342 29091967 PMC5665544

[B8] Candini M. Farinelli M. Ferri F. Avanzi S. Cevolani D. Gallese V.. (2016). Implicit and explicit routes to recognize the own body: evidence from brain damaged patients. Front. Hum. Neurosci. 10, 405. 10.3389/fnhum.2016.00405 27630550 PMC5006097

[B9] Carlson E. B. Putnam F. W. (1993). An update on the dissociative experiences scale. Dissociation: progress in the dissociative disorders. 10.1037/t86316-000

[B10] Cash T. F. Deagle I. I. I. E. A. (1997). The nature and extent of body-image disturbances in anorexia nervosa and bulimia nervosa: a meta-analysis. Int. J. Eating Disorders 22, 107–126. 10.1002/(SICI)1098-108X(199709)22:2andlt;107::AID-EAT1andgt;3.0.CO;2-J 9261648

[B11] Cohen J. (1969). Statistical Power Analysis for the Behavioral Sciences. New York, NY: Academic Press.

[B12] Conson M. Mazzarella E. Trojano L. (2013). Developmental changes of the biomechanical effect in motor imagery. Exp. Brain Res. 226, 441–449. 10.1007/s00221-013-3456-x 23455729

[B13] Cuzzolaro M. Vetrone G. Marano G. Garfinkel P. (2006). The body uneasiness test (but): development and validation of a new body image assessment scale. Eating Weight Disorders Stu. Anorexia Bulimia Obesity 11, 1–13. 10.1007/BF03327738 16801740

[B14] Decety J. Jeannerod M. Germain M. Pastene J. (1991). Vegetative response during imagined movement is proportional to mental effort. Behav. Brain Res. 42, 1–5. 10.1016/s0166-4328(05)80033-6 2029340

[B15] Derogatis L. R. Cleary P. A. (1977). Confirmation of the dimensional structure of the SCL-90: a study in construct validation. J. Clin. Psychol. 33, 981–989. 10.1002/1097-4679(197710)33:4andlt;981::AID-JCLP2270330412andgt;3.0.CO;2-0

[B16] Devue C. Collette F. Balteau E. Degueldre C. Luxen A. Maquet P.. (2007). Here I am: the cortical correlates of visual self-recognition. Brain Res. 1143, 169–182. 10.1016/j.brainres.2007.01.055 17306235

[B17] Esposito R. Cieri F. di Giannantonio M. Tartaro A. (2018). The role of body image and self-perception in anorexia nervosa: the neuroimaging perspective. J. Neuropsychol. 12, 41–52. 10.1111/jnp.12106 27220759

[B18] Fairburn C. G. Beglin S. J. (2008). Eating disorder examination questionnaire. Cognit. Behav. Ther. Eating Disorders 309, 313.

[B19] Faul F. Erdfelder E. Lang A. G. Buchner A. (2007). G^*^ Power 3: A flexible statistical power analysis program for the social, behavioral, and biomedical sciences. Behav. Res. Methods 39, 175–191. 10.3758/BF03193146 17695343

[B20] Ferri F. Frassinetti F. Ardizzi M. Costantini M. Gallese V. (2012). A sensorimotor network for the bodily self. J. Cognit. Neurosci. 24, 1584–1595. 10.1162/jocn_a_00230 22452562

[B21] Ferri F. Frassinetti F. Costantini M. Gallese V. (2011). Motor simulation and the bodily self. PloS ONE 6, e17927. 10.1371/journal.pone.0017927 21464959 PMC3064658

[B22] Ferroni F. Gallese V. (2023). “Social bodily self: Conceptual and psychopathological considerations,” in The Routledge Handbook of Bodily Awareness, eds Adrian J. T. (London and New York: Routledge Taylor and Francis Group), 523–541.

[B23] Franchak J. M. (2017). Exploratory behaviors and recalibration: What processes are shared between functionally similar affordances?. Attention Percept. Psychophys. 79, 1816–1829. 10.3758/s13414-017-1339-0 28547681

[B24] Franchak J. M. Somoano F. A. (2018). Rate of recalibration to changing affordances for squeezing through doorways reveals the role of feedback. Exp. Brain Res. 236, 1699–1711. 10.1007/s00221-018-5252-0 29623380

[B25] Gallagher S. (2000). Philosophical conceptions of the self: implications for cognitive science. Trends Cognit. Sci. 4, 14–21. 10.1016/S1364-6613(99)01417-5 10637618

[B26] Gallese V. Sinigaglia C. (2010). The bodily self as power for action. Neuropsychologia 48, 746–755. 10.1016/j.neuropsychologia.2009.09.038 19835895

[B27] Gardner R. M. Brown D. L. (2014). Body size estimation in anorexia nervosa: a brief review of findings from 2003 through 2013. Psychiatry Res. 219, 407–410. 10.1016/j.psychres.2014.06.029 25023364

[B28] Gaudio S. Quattrocchi C. C. (2012). Neural basis of a multidimensional model of body image distortion in anorexia nervosa. Neurosci. Biobehav. Rev. 36, 1839–1847. 10.1016/j.neubiorev.2012.05.003 22613629

[B29] Gentilucci M. Daprati E. Gangitano M. (1998). Right-handers and left-handers have different representations of their own hand. Cognit. Brain Res. 6, 185–192. 10.1016/S0926-6410(97)00034-7 9479070

[B30] Ghisi M. Flebus G. B. Montano A. Sanavio E. Sica C. (2006). L'adattamento italiano del BDI-II [Italian adaptation of BDI-II]. Beck Depression Inventory-II. Firenze, IT: Organizzazioni Speciali.

[B31] Giannini M. Pannocchia L. Dalle Grave R. Muratori F. Viglione V. (2008). EDI-3 Eating Disorder Inventory-3: Manuale. Firenze: OS Organizzazioni Speciali.

[B32] Glashouwer K. A. van der Veer R. M. Adipatria F. Jong d. e. Vocks P. J. (2019). The role of body image disturbance in the onset, maintenance, and relapse of anorexia nervosa: a systematic review. Clin. Psychol. Rev. 74, 101771. 10.1016/j.cpr.2019.101771 31751876

[B33] Guardia D. Lafargue G. Thomas P. Dodin V. Cottencin O. Luyat M.. (2010). Anticipation of body-scaled action is modified in anorexia nervosa. Neuropsychologia 48, 3961–3966. 10.1016/j.neuropsychologia.2010.09.004 20833193

[B34] Haggard P. Taylor-Clarke M. Kennett S. (2003). Tactile perception, cortical representation and the bodily self. Current Biol. 13, R170–R173. 10.1016/S0960-9822(03)00115-5 12620204

[B35] Ionta S. Fourkas A. D. Fiorio M. Aglioti S. M. (2007). The influence of hands posture on mental rotation of hands and feet. Exp. Brain Res. 183, 1–7. 10.1007/s00221-007-1020-2 17643238

[B36] Jeannerod M. Pacherie E. (2004). Agency, simulation and self-identification. Mind Lang. 19, 113–146. 10.1111/j.1468-0017.2004.00251.x

[B37] Keizer A. Smeets M. A. Dijkerman H. C. Uzunbajakau S. A. van Elburg A. Postma A.. (2013). Too fat to fit through the door: first evidence for disturbed body-scaled action in anorexia nervosa during locomotion. PLoS ONE 8, e64602. 10.1371/journal.pone.0064602 23734207 PMC3667140

[B38] Kosslyn S. M. DiGirolamo G. J. Thompson W. L. Alpert N. M. (1998). Mental rotation of objects versus hands: Neural mechanisms revealed by positron emission tomography. Psychophysiology 35, 151–161. 10.1111/1469-8986.3520151 9529941

[B39] Kosslyn S. M. Ganis G. Thompson W. L. (2001). Neural foundations of imagery. Nat. Rev. Neurosci. 2, 635–642. 10.1038/35090055 11533731

[B40] Longo M. R. Haggard P. (2010). An implicit body representation underlying human position sense. Proc. Nat. Acad. Sci. 107, 11727–11732. 10.1073/pnas.1003483107 20547858 PMC2900654

[B41] Meregalli V. Tenconi E. Madan C. R. Som,à E. Meneguzzo P. Ceccato E.. (2023). Beyond body image: what body schema and motor imagery can tell us about the way patients with anorexia nervosa experience their body. Psychiatry Clin. Neurosci. 77, 94–101. 10.1111/pcn.13501 36330847

[B42] Mibu A. Kan S. Nishigami T. Fujino Y. Shibata M. (2020). Performing the hand laterality task does not necessarily require motor imagery. Sci. Rep. 10, 5155. 10.1038/s41598-020-61937-9 32198401 PMC7083854

[B43] Oldfield R. C. (1971). The assessment and analysis of handedness: the Edinburgh inventory. Neuropsychologia 9, 97–113. 10.1016/0028-3932(71)90067-4 5146491

[B44] Parsons L. M. (1994). Temporal and kinematic properties of motor behavior reflected in mentally simulated action. J. Exp. Psychol. Hum. Percept. Perf. 20, 709. 10.1037/0096-1523.20.4.709 8083630

[B45] Pastore R. E. Scheirer C. J. (1974). Signal detection theory: considerations for general application. Psychol. Bullet. 81, 945. 10.1037/h0037357

[B46] Pedrabissi L. Santinello M. (1989). Verifica della validità dello STAI forma Y di Spielberger. Berlin: Giunti Organizzazioni Speciali.

[B47] Porro C. A. Francescato M. P. Cettolo V. Diamond M. E. Baraldi P. Zuiani C.. (1996). Primary motor and sensory cortex activation during motor performance and motor imagery: a functional magnetic resonance imaging study. J. Neurosci. 16, 7688–7698. 10.1523/JNEUROSCI.16-23-07688.1996 8922425 PMC6579073

[B48] Scarpina F. Bastoni I. Villa V. Mendolicchio L. Castelnuovo G. Mauro A.. (2022). Self-perception in anorexia nervosa: when the body becomes an object. Neuropsychologia 166, 108158. 10.1016/j.neuropsychologia.2022.108158 35033502

[B49] Schwoebel J. Coslett H. B. (2005). Evidence for multiple, distinct representations of the human body. J. Cognit. Neurosci. 17, 543–553. 10.1162/0898929053467587 15829076

[B50] Stefanile C. Matera C. Pisani E. (2009). Body shape questionnaire (BSQ-14): an italian version. J. Eating Disorders 6, 485–494.36429834

[B51] The Jamovi Project (2021). Jamovi. Available online at: https://www.jamovi.org

[B52] Urgesi C. Fornasari L. Faccio D. Perini S. Mattiussi L. Ciano E.. (2011). Body schema and self-representation in patients with bulimia nervosa. Inte. J. Eating Disorders 44, 238–248. 10.1002/eat.20816 20186715

[B53] Urgesi C. Moro V. Candidi M. Aglioti S. M. (2006). Mapping implied body actions in the human motor system. J. Neurosci. 26, 7942–7949. 10.1523/JNEUROSCI.1289-06.2006 16870739 PMC6674209

[B54] Vannuscorps G. Pillon A. Andres M. (2012). Effect of biomechanical constraints in the hand laterality judgment task: where does it come from?. Front. Hum. Neurosci. 6, 299. 10.3389/fnhum.2012.00299 23125830 PMC3485652

[B55] Wraga M. Shephard J. M. Church J. A. Inati S. Kosslyn S. M. (2005). Imagined rotations of self versus objects: an fMRI study. Neuropsychologia 43, 1351–1361. 10.1016/j.neuropsychologia.2004.11.028 15949519

[B56] Yasuda M. Wagman J. B. Higuchi T. (2014). Can perception of aperture passability be improved immediately after practice in actual passage? Dissociation between walking and wheelchair use. Exp. Brain Res. 232, 753–764. 10.1007/s00221-013-3785-9 24306437

[B57] Zacks J. M. (2008). Neuroimaging studies of mental rotation: a meta-analysis and review. J. Cognit. Neurosci. 20, 1–19. 10.1162/jocn.2008.20013 17919082

